# High incidence of respiratory viruses in critically ill adult patients with respiratory failure

**DOI:** 10.1186/cc12073

**Published:** 2013-03-19

**Authors:** M Sietses, TE Faber, L Bont, H Buter, EC Boerma

**Affiliations:** 1 Medical Center Leeuwarden, the Netherlands; 2 University Medical Center Utrecht, the Netherlands

## Introduction

Laboratory testing for viral infections is not routinely performed in adult patients admitted to the ICU. However, reports indicate that viruses may contribute to significant morbidity and mortality and that viral-bacterial co-infection is associated with poor outcome in this particular patient population. The aim of the study was to document the incidence of respiratory virus infections in critically ill adult patients admitted to the ICU for acute respiratory failure.

## Methods

The prospective, observational study took place during two consecutive winter seasons in a mixed 16-bed ICU at the Medical Center Leeuwarden, the Netherlands. Adult patients admitted to the ICU, suspected of respiratory failure due to community-acquired pneumonia, were included. After informed consent brushed nasopharyngeal swab (Copan^®^) samples were taken within 24 hours after admission and analyzed by PCR assays for the presence of respiratory syncytial virus (RSV), influenza virus, metapneumovirus (MPV), parainfluenza virus, adenovirus, rhinovirus and coronavirus. Data are presented as median (IQR).

## Results

Thirty-four patients were enrolled. Median age was 66 (54 to 71) years; 65% of the patients were male; median APACHE IV score on admission was 76 (61 to 96). Median ICU stay was 8 (5 to 17) days, with an overall hospital mortality of 12%. Thirteen patients (38%) tested positive for a respiratory virus. The most frequently found virus was influenza (37%), followed by RSV (15%), rhinovirus (15%), MPV (15%), corona virus (9%) and parainfluenza virus (9%). Two of the patients (6%) had a bacterial-viral co-infection (blood culture and PCR positive for Streptococcus pneumonia and rhinovirus in one patient and for Haemophilus influenza and influenza virus in the second patient). Length of stay in the ICU was significantly longer in PCR-negative patients, in comparison with PCR-positive patients (11 (6 to 20) vs. 7 (2 to 8) days, respectively, *P3<*0.05).

## Conclusion

Respiratory viruses, and particularly influenza virus, are frequently found in adult patients with respiratory failure admitted to the ICU.
